# Teaching LGBT+ Health and Gender Education to Future Doctors: Implementation of Case-Based Teaching

**DOI:** 10.3390/ijerph18168429

**Published:** 2021-08-10

**Authors:** Hsing-Chen Yang

**Affiliations:** Graduate Institute of Gender Studies, Kaohsiung Medical University, Kaohsiung 80708, Taiwan; yhc@kmu.edu.tw

**Keywords:** case-based teaching, clinical psychiatric education, competency learning, gender education, LGBT+ healthcare

## Abstract

Improving the education of medical students and physicians can address the disparities in LGBT+ (lesbian, gay, bisexual, transgender, and others) health care. This study explored how teachers used case-based teaching to teach medical students about gender and LGBT+ health care and discussed the implementation and effectiveness of case-based teaching from the perspective of the teachers and students. This study employed the case study method and collected data through semi-structured interviews. This study used two gender courses in clinical psychiatric education as case studies. Two teachers and 19 medical students were recruited as participants. The findings of this study were as follows: (1) effective cases links theory to clinical practice and competency learning; (2) experience sharing by LGBT+ is highly effective; (3) discussions promote the effectiveness of case-based teaching; and (4) the challenges of case-based teaching included time limitations, the multiplexity of the cases, and multilevel learning. This study also found that using narrative cases is a form of narrative pedagogy, which can help students to integrate medicine, gender, and LGBT+ competency education. A successful narrative case–based teaching strategy involves teachers integrating knowledge related to gender, guiding students through the cases to understand the importance of these cases, and reflecting on the medical profession to make improvements. However, teachers face challenges in this approach, such as changes in the school’s teaching culture and a lack of institutional support.

## 1. Introduction

In a heteronormative society, lesbian, gay, bisexual, transgender, and others (LGBT+) community members face considerable challenges regarding sexuality and gender identity. Additionally, they are often victims of sexual and gender-based violence and discrimination [1,2,3,4]. Moreover, their sexuality and gender identity can affect their medical rights and the medical treatment they receive, and their medical needs are more likely to be neglected or socially rejected [2,3,4]. For example, when consulting with medical personnel, LGBT+ often encounter the problem of coming out. Their worry is twofold. First, their medical rights can be affected by the insufficient knowledge, prejudice, or discrimination of medical personnel. Second, insufficient disclosure of information can affect the diagnosis of a disease and even result in misdiagnosis [2,5,6]. LGBT+ must carefully weigh the risk of coming out and the benefit of receiving appropriate medical care and resources. Such considerations often create psychological pressure, which adversely affects physical and mental health. These are some of the health disparities and inequalities that the LGBT+ faces. 

Gender issues have long been a focus of psychiatry, especially issues regarding LGBT+ sexuality, gender identity, and physical and mental health [7]. Psychiatrists should be equipped with gender competence to understand the potential mental distress related to gender and provide the appropriate health care and treatment to patients. Competence refers to an integral ability based on relevant knowledge, attitudes, and skills that learners acquire from education; they enable the handling of complex personal or social lives and challenges [8]. When exhibited in professional medical fields, gender competence, according to Yang and Yen’s definition, results from the collective integration of learning gender knowledge, attitude, and skills that can enable a physician to apply appropriate clinical practices and professional actions to gender-friendly healthcare [9].

Although gender competence is generally acknowledged as an essential professional competence for doctors [10,11,12], it does not come into being suddenly to be added to a doctor’s set of professional skills. Rather, doctors must be empowered through gender education. Gender education prepares physicians to comprehend topics related to LGBT+ health and gender, such as discrimination; gender-based violence; sexual orientation; gender identity; and mental, behavioral, and sexual health. Medical education in universities and colleges is a form of professionalization and resocialization, and the mainstreaming of gender education in medical education emphasizes the importance of gender awareness and gender education [13,14].

Curricula and teaching are inextricably linked. Teaching methods are crucial to effectively implement any education. Using the appropriate teaching methods is key for teachers to educate successfully and improve the effectiveness of students’ learning. Therefore, this study examined courses on gender for psychiatric clinical practice to investigate the use of case-based teaching in LGBT+ health care education and the experience of teachers and medical students using this method. The results can offer teachers strategies to implement case-based teaching in gender and medical courses, which can improve LGBT+ health care.

### 1.1. LGBT+ Health Care and Gender Education 

Social systems are closely related to culture and human health. Numerous studies have demonstrated that the social problems faced by LGBT+ people, such as stigma, homophobia, discrimination, coming out, weak social support systems, and insufficient LGBT+ friendly medical resources, are not only likely to cause physical and mental harm but also constitute risk factors that endanger health [3,4,15,16,17,18]. The discrimination, violence, and bullying that LGBT+ may encounter in the process of discovering their gender identity represent threats to physical and mental health. This type of minority stress can increase the vulnerability of LGBT+ people, leading to mental health problems, such as anxiety and depression [3].

In addition, because the mainstream discourse on sexual health and sex education reflects heteronormativity, which considers heterosexuality the “normal” sexuality, medical staff may not fully understand the sexual health issues in LGBT+ people [19]. Marrazzo et al. [20] discovered that some members of the lesbian community were told by medical staff that cervical screening was unnecessary because they had not had sexual intercourse with men. However, the fact that members of the lesbian community are sexually attracted to women does not mean that they have never had sexual contact with men. Another study documented the experience of a patient assigned female at birth in deciding to transition; because she worried that she would be unable to provide sufficient evidence, she deliberately accentuated her masculine side and said what she thought the doctor wanted to hear to earn the trust of the doctor. In a society based on a binary perspective on gender, if doctors lack sensitivity and competence in issues related to gender, such as transgender issues, those who wish to transition cannot be true to themselves and often feel compelled to deceive doctors to receive their trust [21]. 

Many studies have indicated that improving medical education and incorporating LGBT+ health care issues into medical education and training are necessary to advance health equity for LGBT+ [3,4,22,23]. One study introduced a half-day compulsory teaching program to a large medical school in London. The program was conducted through collaboration with the LGBT+ community and increased the students’ confidence in using the appropriate language for topics related to sexual orientation and gender identity and for the clinical assessments of LGBT+ patients [4]. One study demonstrated that an LGBT+ health equity curriculum changed medical students’ implicit attitudes and biases toward LGBT+ people and highlighted the importance of providing time to debrief and explore implicit biases [24].

Studies have also highlighted the need to reform medical education with competency-based education [25,26,27]. Several studies have emphasized that medical students can obtain the knowledge, attitudes, and skills required for LGBT+ competence only through improvements to teaching methods [3,25,26]. One study reported that the LGBTQ Health Pathway program developed by a student-driven curriculum enables medical students to enhance their training in the care of LGBT+ patients. It also reported that the LGBTQ program enhanced medial students’ interest in LGBT+-related medicine [28]. One study reported that increasing the amount of LGBT+ teaching in undergraduate medical curricula could increase medical students’ awareness of LGBT health issues and increase positive attitudes towards LGBT+ patients [29].

However, incorporating LGBT+ health content into undergraduate medical curricula is challenging because the appropriate pedagogical methods to teach this material have not been identified [26]. Sekoni et al. [22] noted that developing teaching methods on the basis of educational theory could elucidate “why certain teaching approaches work well for particular groups and contexts.” Teaching methods are crucial to successfully incorporate LGBT+ health issues into medical education and develop students’ competence in LGBT+ issues.

### 1.2. Case-Based Teaching 

Teaching is a design science [30]. It requires the development of a teaching plan. The appropriate teaching method can be selected by evaluating a teacher’s grasp of the contents and goals of education, considering the characteristics and learning needs of the students and incorporating supplemental assessments. The contents and activities must also be presented in a manner that the students can easily understand [31].

Case-based teaching is based on constructivism and situational learning theory. In this method, students apply what they learn to real-life problems through a multidisciplinary learning framework. The method deepens students’ professional knowledge and trains them to apply their knowledge to analyze and solve problems [32]. Case-based teaching is a practice-based, problem-solving-oriented method of teaching. The cases are used as the teaching materials and combined with the topics of the course. The method involves teacher–student interactions, such as discussions and question-and-answer sessions regarding the context and problems of a particular case, that provide students with an understanding of the theories and knowledge related to a certain topic. Students are trained in analysis, reflection, and critical thinking and develop problem-solving skills and the ability to achieve interdisciplinary coherence [33,34,35,36,37,38]. 

Practicing doctors should possess excellent judgment and practical knowledge, which cannot be obtained through oral lectures given by teachers alone [37,39]. Case-based teaching trains medical students on both the know-what and the know-how (such as diagnosis and intervention) and develops their clinical problem-solving skills [37,40,41,42]. Minturn et al. [43] developed a 10-h LGBT+ health curriculum, including case-based small-group discussions covering LGBT+ terminology for preclinical medical and physician assistant students. They reported that the curriculum was effective at improving medical students’ self-confidence in working with LGBT+ patients, even though it proved less effective at increasing LGBT+ related medical knowledge.

Studies have indicated that medical students believe that case-based teaching is more effective than traditional teaching methods for acquiring knowledge and improving problem-solving skills [39,40,44]. Several studies have demonstrated that medical students generally agree that case-based teaching is an innovative educational approach for acquiring knowledge. The teaching method allows students to apply their knowledge to cases and develops their critical thinking and analysis skills, which can contribute to future clinical practice [33,37,41,42,45].

The case method can be implemented by knowing what questions to ask, when to ask them, and how to structure them to effectively facilitate problem-solving discussions [46,47]. The types of questions that can be used to present a case are as follows: (1) opening questions: posed at the beginning to pinpoint the key topics in the case, collect information relevant to the case, and identify the problems that occurred in the case; (2) analytic questions: posed to prompt students to analyze the case; (3) evaluative questions: posed to prompt students to use their critical thinking skills to determine the appropriate method to address the case; and (4) synthesis: posed to emphasize critical holistic topics (e.g., “How have prior actions affected, expanded, or limited the available options?”, “What method would you use to solve this problem and what are its advantages?”, and “What are potential drawbacks to consider?”) [46,47]. 

Case-based learning facilitates the development of learning communities and can develop students’ abilities to debate, think critically, perform multifaceted analyses of problems, and conduct problem-solving. The case-based method is a useful tool for teachers to educate medical students and future physicians about LGBT+ health and medical care.

## 2. Methodology

In this study, a case study was conducted and data were collected through in-depth interviews. The case study method involves collecting and analyzing data for a specific individual, group, or event, in order to gain in-depth understanding of the specific case, including a greater understanding of the phenomenon or problem, and then finding a solution or new perspective. A good case study can help researchers grasp and present insights at the micro and macro level; in addition, it has the effect of echoing and improving theory or practice [8,48].

Two courses on gender for clinical psychiatric education were developed in collaboration with the affiliated hospital of Kaohsiung Medical University (KMU). The two courses were used as cases studies. Both teachers and students in medical education were recruited as participants to explore the application of case-based instruction and its effects on LGBT+ health and gender education.

### 2.1. Research Setting and Participants

In Taiwan, medical education is divided into three phases: undergraduate medical education (basic medical education and clinical education), the postgraduate year program (PGY), and continued medical education (specialty development). In the 6-year undergraduate medical education program, basic medical education is provided in the first 4 years, and clinical education is provided in the final 2 years [8].

This study was conducted at KMU, a long-established medical university in Taiwan with affiliated medical centers and hospitals. Clinical psychiatric education in KMU lasts 1 month, during which time interns participate in morning meetings, rounds, team meetings, and attend core curriculum tutorials. The core curriculum comprises six psychiatric clinical topics, each of which lasts 1 h, and the number of students per session is 8–12. Two new courses on gender were added to the students’ intern training in clinical psychiatric education. The courses were entitled “LGBT+ Health and Medical Care” (LGBT+ HMC) and “Gender Violence and Gender Dysphoria.” 

These gender-related courses were held at separate times, with each session lasting 2 h, enabling each student to participate in both courses. Two attending physicians were responsible for teaching the two courses, and each was qualified as a psychiatrist and held the position of medical university professor. A total of 230 medical students attended the psychiatric clinical education training from September 2017 to May 2018. Among them, 19 students (11 fifth-year and eight sixth-year students; 11 men and eight women) were interviewed for this study. In short, 21 informants participated in this study, including two teachers (psychiatrists) and 19 medical students (Table 1).

### 2.2. Instructional Design and Implementation of the Courses

The two gender courses were based on competency-based learning, and knowledge, attitudes, and skills were used as the core components [8]. The contents and objectives of the LGBT+ HMC course were as follows: (1) to understand how social structures, culture, and other factors influence the lives of LGBT+ people; and (2) to understand how sexual orientation, gender identity, gender expression, and gender discrimination affect the physical and mental health of LGBT+ patients. The Gender Violence and Gender Dysphoria course focused on the following topics and instructional objectives: (1) to acknowledge the existence of various forms of gender violence and the interaction between gender violence and the recurrence or exacerbated symptoms of mental illness; (2) to understand the mental harm or psychological abuse caused by violence committed by an intimate partner and its negative effect on physical and mental health; (3) to comprehend the concept of gender dysphoria, empathize with transgender individuals, and understand their expression of gender; (4) to learn about sex reassignment surgery and the regulations, referrals, and resources related to it; and (5) to understand how transgender men and women can differ in terms of subjective and objective distress and overall prognosis.

The two courses had the same instructional design, which consisted of warm-up activities, concept introduction, cases and activities, and a conclusion. For the warm-up activities, the LGBT+ HMC course used a 3 × 3 square game consisting of nine questions. The game was both a warm-up activity and a learning assessment tool [8]. 

Three types of materials were used in the case-based teaching: (1) written cases: cases adapted from notorious incidents related to gender-based violence in Taiwan that allowed students to understand the effect of gender-based violence on mental health; (2) narrative films: life stories of transsexual people were presented to students to help them understand gender dysphoria, transgenderism, and sex reassignment; (3) LGBT+ people sharing: two LGBT+ persons were invited to share their personal life stories (focusing on the influence of their LGBT+ identity on their physical and mental health and interpersonal interactions), describe their medical needs, and detail some of the medical difficulties they had encountered. Gender and LGBT+ health care issues in patient–doctor interactions were also discussed.

### 2.3. Data Collection, Analysis, and Research Ethics

Data were collected through semi-structured, in-depth interviews with teachers and students, separately. The duration of each interview was approximately 1–2 h, and each interviewee was interviewed one or two times. Before the interview was conducted, this study provided an interview outline for the interviewee in advance and explained that the interview outline was only a guide for the interview. The interviewee could modify the interview outline or raise other questions for discussion on the subject matter of this study. Although the interview outline for teachers and students differed, the interview outline basically consists of two parts: (1) warm-up questions: for example, asking the interviewee to share his/her experiences of teaching/learning in the field of clinical education; past related teaching/learning experiences on gender courses; (2) main questions: the interviewees were asked to discuss their feedback on the two courses, such as teaching/learning experience and reflection, feedback on case-based teaching methods, and the evaluation of teaching/learning performance and outcome. 

In the actual interviews, this study followed the principle of semi-structured interviews and flexibly adjusted the order of questions and the content of the interviews based on actual interview interactions. The interview place was chosen to be quiet and peaceful, comfortable and convenient for the interviewee. With the consent of the interviewee, the audio of each interview was recorded, and notes were taken. The interviews and the classroom observation notes were transcribed and used for data analysis. The observation notes were recorded by the researcher and the research assistants and detailed each lesson and the interactions between the teacher and the students during class. 

The two courses were implemented for 1 academic year. Because of concerns regarding research ethics, the participants were invited to voluntarily participate in the interviews only after they had completed the courses and the academic year had ended. Therefore, the students were aware that their participation in this study was irrelevant to the results of their internships. Many of the students received invitations to participate approximately 1 year after the end of the courses; some had graduated and left to begin their PGY training, and others were preparing for national examinations. Because of ethical considerations, the design of the study, and factors related to time and distance, a total of 19 students were able to participate in the study.

The data were coded to the identity of the participant and the course code. In the example of code S0101, the first three digits (S01) refer to the first student, and the last two digits (01) refer to the Gender Violence and Gender Dysphoria course. The same rules apply to T0102, in which T01 denotes the first teacher and 02 represents the LGBT+ HMC course.

A thematic analysis was used, and four themes were identified from the interviews as follows: (1) effective cases link theory to clinical practice and competency learning; (2) experience sharing by LGBT+ is highly effective; (3) discussions promote the effectiveness of case-based teaching; and (4) the challenges that emerged were due to time limitations, the multiplexity of the cases, and multilevel learning. 

In terms of research ethics, informed consent was received after the participants were informed that they could withdraw from the study and that the audio of the interviews were recorded and transcribed. To ensure that the research was ethical and anonymous, the interview data were coded and revised to enhance the readability of the sentences, but the speaker’s intended meaning was retained.

## 3. Results

### 3.1. Effective Cases Link Theory to Clinical Practice and Competency Learning 

Traditional lectures are the most commonly used teaching method in the school in this case study. In an examination-oriented educational environment, the teacher’s instruction focuses on helping students pass the national examination to obtain a medical license. However, the disadvantage of the lecture method is that it is a one-way channel of knowledge exchange and lacks teacher–student interaction. In disease-oriented lectures, a disease is explained in terms of its pathophysiology, clinical manifestations, diagnosis, examination, medical treatment, and health care process; decontextualized knowledge transfer is unlikely to engage students [49]. Although teachers recognize the importance of LGBT+ health care education, they worry that students may be uninterested because these issues do not appear on the exam.

“*Gender may be less important to students because they need to study for tests. Because students must prepare for the national exam, they tend to be test oriented. They cannot accomplish anything without a license, which causes them to neglect gender issues because they seem irrelevant to the national exam*”.

(T0201)

The findings revealed that medical teachers in clinical education are under pressure to assist medical students in passing the national examination and acquire a medical license. In such an educational environment, the success of case-based teaching is closely related to the quality of the case [32]. Therefore, using case materials that impress students and stimulate their interest is crucial. Story-based cases, whether in the form of writing, film, or experience-sharing, are motivational teaching materials that can encourage students to learn.

“*I was worried that the students would not be interested in the courses...The cases you provided are great, and the teaching plan and activities are great too... What was surprising was that the students actually raised questions themselves and were interested in the topics*”.

(T0201)

The teachers indicated that, unlike conventional lectures, in which students remain silent and unresponsive, the courses on gender and case-based teaching enabled students to demonstrate their interest in learning about gender and LGBT+ health and motivated them to engage in learning. The interviews with the students yielded similar results.

“*The teacher gave us a detailed case that I had never heard of before for us to study in class. The case allowed us to sympathize with the character in the story, which made us aware of gender-based violence and the effects it can have*”.

(S0501)

“*The case went deep into the story and described situations that were difficult to imagine for us as non-LGBT+ people. The case study allowed us to experience something that rarely occurs in our daily life. We wouldn’t have known about gender-based violence if it wasn’t for the case-based learning. When the story came to an end, I realized that gender-based violence can also strongly affect an individual’s mental health*”.

(S1501)

The feedback from students indicated that cases motivated the students to learn and drew their attention toward gender issues. By engaging with the stories, the students expanded their understanding of gender issues in their real-life interpersonal relationships, the embeddedness of gender in society and social relations, and the impact on physical and mental health.

The students responded that, compared with past medical education courses, the courses on gender “*were entirely different in that we learned by studying several cases* (S0301).” The majority of students noted that they “*do not have a strong impression*” and “*do not remember much*” about the concepts related to gender and psychiatry. However, they reacted positively to the cases and the case-based learning, stating that “*I only remembered that part*,” “*the* *case study was my favorite part*,” and “*I understood the life of an LGBT person by watching the film.*”

Case-based teaching is a more effective method of acquiring knowledge than traditional teaching, and it made the subjects easier to learn and solidified the students’ understanding of these subjects [41]. One shortcoming of knowledge-oriented education is that the knowledge acquired is separated from its context, creating a gap between theory and practice. This division makes students unable to apply knowledge and put it into practice. This study revealed that case-based teaching could encourage students to be actively involved in the process of learning and motivate them to develop an interest in a subject. Case-based teaching also helped students make the connection between knowledge and situated learning and provided a real-life context for students to acknowledge the tangible connection between gender and psychiatry. 

The interviews with the teachers and students demonstrated that case-based learning motivates students to learn about gender and pay more attention to LGBT+ health issues. Effective cases can deepen students’ understanding of LGBT+ health issues in their real-life relationships and their understanding of how gender functions in social frameworks and relationships by affecting physical and mental health.

### 3.2. Sharing LGBT+ Stories Is Effective 

The teachers who participated in this study admitted that, before giving the courses, they were worried about the students’ acceptance of the courses and LGBT+ issues. However, after the classes had ended, they stated, “*The courses were well-designed, and hearing transgender people share their experiences generated some great discussions*... *many of the students responded well! The students’ responses to the lecture showed that they were truly interested in the topic. Some people asked the lecturer questions after class, and some even continued to discuss the topic after the class was over*” (T0201).

Almost every student mentioned that hearing the LGBT+ speakers share their experiences, including their experiences with health care, was their favorite part of the course. They indicated that “*this almost never happens in other courses”* (S0501) and that they were awestruck by the experience:

“*I really think that inviting transgender people to share their experience is valuable. It makes an impression when you see a person standing in front of you speaking… it’s really striking. It was a good experience, and we got to ask questions afterward*”.

(S0301)

“*A lesbian nurse was invited to class to share her experience. The sharing session was quite astonishing, especially for students who had never met LGBT+ people, and it must have had a subtle effect on those students and allowed them to learn something. Experience sharing is unique because we get to listen to other people’s lives and true feelings that you can’t find in books, which helps us develop empathy and gain insight. I walked away being able to say ‘this is how the patient may be thinking’ and ‘this is how we can treat the patient.’ The sharing session changed me in terms of my clinical practice. For example, I’ll think to myself, ‘Oh! I can educate LGBT+ people about a certain health topic,’ or I’ll pay more attention to my choice of words when interacting with LGBT+ people*”.

(S1402)

In addition to stimulating the students’ interest in learning and their emotional responses, the sharing of real-life stories enabled the students to sympathize with others. The experiences and stories shared by the LGBT+ people demonstrated the effects of social norms and patriarchal systems on an individual’s life, physical and mental health, and access to health care services. This study revealed that using highly interactive narrative texts allowed the teachers to achieve teaching and learning goals effectively and facilitated the incorporation of attitudes and skills into learning, as indicated by one teacher:

“*Although we emphasize thinking from the patient’s perspective, we rarely ask patients to describe their problems and difficulties from their perspective. Listening to others share their experiences helps us solve that problem and encourages students to reflect on the kind of pain their patient is actually experiencing, rather than making assumptions. This can have a strong effect on the students*”.

(T0201)

The students and teachers are noted differences between watching films and hearing members of the LGBT+ community share their experiences:

“*Hearing a member of the LGBT+ people share their experiences might be better. Watching a film sometimes makes people sleepy, but hearing someone share their experiences does not because of the direct interaction and Q&A session with the speaker. We’re also able to ask questions directly to the speaker*”.

(S1602)

“*Seeing a concert in person is different from watching it on TV, which is less direct. Films may still be effective, but to me they lack a sense of presence. Hearing a member of the LGBT+ people share their experience lets you ask questions on the spot, if you have any. You can also interact with them directly. The transgender person that we invited adjusted the way they shared their experience based on the students’ responses. This is a dynamic way of learning, which cannot be achieved by watching films. I believe that films are better than text, but hearing a person share their experiences surpasses both. The interpersonal interaction of sharing experiences leaves a strong impression, which makes it a much quicker way of learning than knowledge-oriented lectures*”.

(T0201)

The difference in the three types of case studies is the fact that having LGBT+ people share their experiences combines the advantages of both written cases and films. The students can interact with the speaker and listen to their voice. The sense of presence the teacher in T0201 described refers to the different experience of each case-based teaching material. The importance of presence lies in its ability to create an environment for affection-based learning that can change attitudes, thereby creating more opportunities for teacher–student interactions and allowing students to ask questions and receive an immediate response. In addition to facilitating discussions and dialogue surrounding a problem, direct interaction enables the educator or speaker to create a class that fits the competency education–immediate feedback paradigm.

Increasing the amount of interaction in education fosters LGBT+ competency among students, changes their attitudes toward LGBT+ people, and deepens their sense of compassion. Such positive changes can improve students’ clinical skills and encourage them to take actions.

“*I never truly considered the thoughts of people with gender dysphoria. I also never considered their emotions. Of course, because they were my patients, I didn’t refuse to treat them. But now, I genuinely want to help. If I ever encounter such patients in the future, I am willing to put all my effort into helping in any way possible in addition to performing the professional procedures for sex reassignment surgery. I really want to help out*”.

(S0101)

“*The lesbian nurse shared her experience as medical staff, for example, how she handles certain situations, and as a patient. I think hearing her share her experience was informative, and personally I learned a lot. I do not discriminate against homosexual people, but I never knew how to educate them about certain health issues. If I did meet a homosexual person, I wouldn’t know what to do, even though I don’t discriminate. The lesbian nurse taught us a lot, including how to handle such situations in a friendlier manner and not cause harm by providing us with specific examples. The experience is pretty helpful for doctors; I learned how to educate such patients about their health*”.

(S1402)

The experience affected the students by changing their attitude and increasing their capacity for emotional empathy and care. The stories shared by the LGBT+ people allowed the students to understand their lives and medical situations, thus fulfilling the objectives of the course.

### 3.3. Discussions Promote the Effectiveness of Case-Based Teaching 

Medical education in Taiwan, including clinical training, is lecture-based; this was the case for the school investigated in this study. One student provided a succinct description: “*Medical education in the past 6 years has been PowerPoint slides only*” (S0301). Because the education system has not changed over time, teachers have encountered numerous challenges. The teachers in this study expressed concerns that students would respond poorly to the courses on gender. 

However, most students responded positively: “*I would like more discussions*” and “*There should have been more discussions*.” Upon further analysis, this study identified two of the students’ opinions regarding the in-class discussions. First, the discussions stimulated the students’ interest in learning about gender and LGBT+ health, and they expressed a desire for more discussions to promote education on gender:

“*The teacher told us to speak up [referring to the case and Q&A session]... it was interactive. We also learned about others’ ideas, and I felt a sense of involvement. If I were to give a suggestion, I would like group discussions in which we can exchange ideas and express our opinions to be incorporated. This sounds like a pretty good idea because sometimes when I hear other people’s ideas, I’ll think to myself, ‘Why didn’t I think of that?’ This is one of those times when I can learn from others. Group discussions can clue me in to how others think and fill in some gaps in my knowledge*”.

(S0101)

Second, the desire to discuss is an opportunity for dialogue. Although many students expressed some disappointment in the lack of depth and time for discussion, such emotional responses were interpreted in this study as being positive, regardless of the emotional implication. Such responses included “*I want group discussions*,” “*There should be more discussion*,” and “*It is not OK to only distribute worksheets without any discussion*,” as well as the following:

“*Concept-based teaching only focuses on delivering a lesson, but you never get specific examples…whereas case studies can bring up multiple topics. You can clearly recognize the causal relationship and understand the pressure that drives someone to exhibit certain behaviors. The case was relatable and allowed me to make connections with real life...maybe people from where I live are more traditionally minded; the majority of them think that a married couple should resolve disputes on their own…but discussions provide answers to my questions (what I wish to understand)…such discussions may seem like we are caring about others, but I am actually responding to my surroundings…*”.

(S1301)

The emotional feedback from the students desiring more discussions reflected their engagement and investment [50,51] in the issues in the courses on gender. These topics related to the students’ lives and encouraged them to start thinking about gender in life and in medicine. Critical thinking and dialogue are required to develop competency-based education, integrate skills, and achieve the goals of the curriculum. Critical thinking and dialogue combine reflection and action. Moreover, dialogues about critical thinking are not monologues [52]. Discussions can start a dialogue, which can facilitate the transformation of ideas and create action. In addition, discussions provide students with the opportunity to practice their clinical skills and decision-making process.

One of the core principles of case-based teaching is discussion [32]. Discussions do not merely involve raising questions and the exchange of ideas among students. Asking questions is key to creating discussions [46]. Knowledge must serve as the scaffold for learning in discussions. The acquisition of knowledge is the basis of critical thinking regarding gender and LGBT+ health issues. The absence of discussion in teaching activities may diminish the effect of concept-based teaching and separate gender knowledge from students’ daily lives. Discussion can generate connections and reduce perceptions of alienation, thereby strengthening cognition, improving attitudes, and facilitating skill-learning. The students reported that the oppressive situation of the character in the case studies resonated strongly with them and affected their processes of medical diagnosis and decision-making. This indicates that discussion improves gender competency in the medical field. 

### 3.4. Challenges: Time Limitations, Multiplex Cases, and Multilevel Learning 

The advantage of case-based teaching is that it applies complex problems and situations to real life, thus providing students with a multifaceted perspective to think critically and explore problem-solving approaches [33,35,37,39]. Under time restrictions, the richness and complexity of the cases can present challenges to instructors, such as uncertainty regarding the topic of discussion during class.

“*The film was well-shot, but there was one problem: people tend to focus on the transsexual person’s wife…people who oppose sex reassignment might say, ‘Look how much harm you’re doing to your wife!’*”.

(T0101)

“*For gender dysphoria, focusing on the surgical procedure is irrelevant to the person’s gender. But, it’s not totally irrelevant. If you delve into the reasons for having sex reassignment surgery, many would say that it’s in the pursuit of gender and sexual identity. During that process, people who are close to the transgender person may be affected. Students may be confused as to whether to discuss this topic from only the perspective of the transgender person or to also consider the opinions of other people, such as their partners.*”.

(T0201)

However, the students explained, “*I learned the most from the part about the surgery, but the teacher did not go into detail about it. I wanted to understand it better, but the information was limited*” (S0301).

The film consists of a person with gender dysphoria recounting his shift in gender identity, the process of transitioning, and the change in the intimate relationship with his wife. Various aspects of gender dysphoria, such as self-identity, social relationships (e.g., interpersonal and intimate relationships), and the medical treatment of transsexual people, can be discussed from the film; the aim is to facilitate LGBT+ health and psychiatric education. The stories in this film aroused students’ learning motivation and interest in deep learning about transgender issues and gender dysphoria. However, the teacher considered that it was still necessary to focus on the protagonist of the film first; otherwise, the teaching focus would be lost.

In contrast with superficial knowledge imparted by traditional lectures, case-based teaching is interdisciplinary and promotes deep understanding and high-level learning. Case-based learning allows students to conceptualize topics and apply their knowledge, whereas lectures prioritize rote memorization [53]. This study discovered that under time constraints, the richness and complexity of the cases necessitated a single, well-defined goal and the application of core knowledge. Otherwise, the focus can easily diverge, and the effectiveness of case-based teaching decreases because of the time limitations and the teacher’s ability and decision to direct the discussion on relevant issues. 

## 4. Discussion

### 4.1. Narrative Cases Integrating Case-Based Teaching and Narrative Pedagogy

This study discovered that high-quality narrative cases are crucial teaching materials to achieve LGBT+ competency through education and develop knowledge, attitudes, and skills. The films, written cases, and experiences of LGBT+ people represented different forms of storytelling. These narrative cases involved the life stories of the LGBT+ people related to both gender issues and psychiatry. Such teaching materials can connect multiple disciplines and facilitate interdisciplinary learning, as described by the students:

“*For one thing, combining gender issues with psychiatric treatment is rather unique…If these concepts of patriarchy, gender-based violence, and other gender-related topics had been taught directly without the inclusion of a narrative case, I might not have attended the class as much. After discussing the case and listening to the teacher’s analysis, I was able to concentrate fully*”.

(S1501)

“*After taking the course, I now have a better understanding of the mental health problems related to gender-based violence. In the case our teacher presented, I was able to identify multiple types of gender-based violence. People who have gone through such experiences may develop mental problems. This case helped me understand the process and allowed me to make connections*”.

(S0101)

These narrative cases not only allowed students to learn about gender-based violence, gender dysphoria, LGBT+ medical care, and physical and mental health at the levels of cognition and attitude but also offered them ideas about how they should take actions. Story-based interdisciplinary cases can offer students opportunities to compare, analyze, and integrate knowledge and can facilitate learning that complements students’ clinical training, thereby achieving the goal of integrated learning.

This study also discovered that using narrative cases is a form of narrative pedagogy that encourages situated cognition and situational leaning. The case stories allowed the medical students to understand the social situations, medical problems, and predicaments of LGBT+ people, which encouraged the transfer of learning and gave students a new perspective and the opportunity to reflect on their roles and responsibilities as a physician through their emotional investment. Narrative teaching prioritizes experiential learning, which can elicit empathy in students. It can also apply abstract theoretical knowledge to real life, which allows students to develop empathy, change their attitudes, and fully comprehend the problems faced by LGBT+ patients in addition to disease.

This study revealed that the stories told by the LGBT+ people were the most effective strategy. Through their stories and medical experience, the students acquired new knowledge and understood the value of their stories for practical applications. The stories were imbued with a sense of realism and meaning when described by the speakers, reflecting the core principle of case-based and narrative teaching, which emphasizes people rather than events. This process allowed the students to understand and emotionally invest in problem-solving methods. Several studies have indicated that inviting LGBT+ to discuss their medical experiences reduces prejudice [4,54] and offers more opportunities for students to interact with the community, which increases students’ awareness. One study indicated that inviting transgender and nonbinary patients to relate their medical experience in person deepened medical students’ understanding of sexual orientation and gender identity and increased their confidence in interacting with such individuals by demonstrating how to use appropriate terms to address transgender people and properly assess their health care needs [4]. 

The LGBT+ speakers invited by this study were nurses. When they shared their medical experience, they described their personal experience as patients and their experience and interactions with other medical staff as medical professionals and LGBT+ people. The speakers offered a multifaceted account of their experience, which allowed the students to view their experiences from different perspectives and reflect on how medical staff interact with LGBT+ patients and provided medical intervention and health care.

Therefore, narrative case teaching is not only sharing stories but also integrating gender perspectives and guiding students to examine the details of a case. The students fully understood the stories and the meaning of the speakers’ experience; they also reflected on their medical knowledge to improve their medical and health care capabilities. This study revealed that narrative case teaching contributes to students’ application of gender education and medical knowledge to LGBT+ health care. This type of education allows students to understand and reflect on experiences to acquire knowledge they can apply clinically to care for patients.

### 4.2. Professional Development for Case-Based Teaching 

The findings of this study are consistent with those of other studies. For example, this study discovered that case-based teaching is an effective method because it motivates students to learn and strengthens their analysis and problem-solving skills. This process increases the effectiveness of education and the students’ levels of satisfaction [36,37,40,41,45,49]. However, case-based teaching entails several challenges for teachers. First, in case-based teaching, cases serve as a vehicle and professional knowledge serves as a reference; the application of professional knowledge to practice is emphasized. Such teaching highlights the complexity of a case [55]. However, this emphasis became a challenge for teachers to implement in this study:

“*You should select certain topics as the take-aways of this course…because the also covered plastic surgery…. Such interdisciplinary teaching is difficult for teachers because requires a great deal of effort to coordinate*”.

(T0201)

To a certain extent, case-based teaching has the spirit of the biopsychosocial education model valued in medical education. However, the application of this model in clinical teaching is difficult, particularly under time constrains. Competency is the goal of interdisciplinary integration. The balance between interdisciplinary knowledge and competency warrants further investigation. 

As Mostert [55] stated, multilevel learning and deep learning are not straightforward procedures. Layers of analysis and progressive discussions are required to reveal the hidden issues and create a learning space for new knowledge and reflection. However, teachers may not have sufficient time to conduct deep learning. Case-based teaching only allows for focus on specific issues and limits the time for discussion. Therefore, reducing the complexity of a case and focusing on specific learning goals within the limited time of a course can help students understand the multiple approaches to analyzing a problem and the essential points of the course.

Second, for teachers accustomed to lecturing, explaining and analyzing the cases to the students through case-based teaching is not particularly challenging. However, guiding students through multilevel dialectical discussions about their questions is challenging. Bauman noted that a perfect world without any accidents, ambiguity, and conflict would be unable to foster moral consciousness, care, mutual assistance, respect, and tolerance in the population [56]. Teachers who wish to develop students’ gender and LGBT+ competence must begin debates about diversity in cognition, attitudes, values, and experience; such developments are achieved by adopting teaching methods that differ from conventional ones, such as case-based teaching and discussions. This study discovered that the instructor’s ability to lead discussions is a key factor that determines learning effectiveness.

In case-based teaching and discussions, the teacher plays the role of a listener, guide, and facilitator. Therefore, in terms of professional development, teachers must improve their ability to use case-based teaching, especially to lead discussions and respond to students’ questions. Because competence-based medical education represents a renaissance in professional education, the professional development of teachers plays a vital role [30]. Competence-based medical education emphasizes participation and innovative teaching, assessment methods, and learning tools, all of which can equip teachers with the required skills and strategies [30,57]. Hence, the professional development of teachers is essential to the successful implementation of case-based teaching. Professional development is also a prerequisite to medical education reform and a responsibility that an educational institution must shoulder.

## 5. Conclusions

Education is the only approach to improving doctors’ gender and LGBT+ competency. Teaching methods are essential to successful education. This study promoted LGBT+ people’s equal right to health by exploring case-based teaching in clinical psychiatric education courses to teach medical students about gender and provide them with knowledge about LGBT+ health care.

This study discovered that case-based teaching could help students understand the social and medical situations faced by LGBT+ people. The case-based teaching allowed students to deeply understand issues regarding gender and equal rights to health and their effect on physical and mental health. For students, case-based learning may serve as a substitute for direct experience, allowing students to understand the situation of a patient beyond the level of a disease. In addition, students can learn through practice-oriented situational problems and develop their reflection, analysis, speculation, and problem-solving skills.

The central tenet of competency-based medical education is the cultivation of high-level competency, which is driven by motivation to learn. The story-based cases encouraged students to learn and actively participate. Narrative cases, particularly the experiences of LGBT+ people, helped create an atmosphere of interaction and opportunities for dialogue, providing students with an environment for deep learning. The results also revealed that case-based teaching is a form of narrative teaching, which can aid students in developing critical thinking, clinical problem-solving, and diagnostic interpretation skills. The life stories and medical experience shared by the LGBT+ speakers helped the students connect professional medical knowledge and clinical experience, thereby strengthening their ability to use their knowledge in clinical applications.

The results also revealed that the discussions facilitated the students’ acquisition of knowledge on gender, increased their emotional investment, integrated theory into practice, and expanded their understanding of the experiences and physical and mental health of LGBT+ people. This is a continuous process of competence learning because the students gradually learned to apply their knowledge to solve the patients’ problems and care for LGBT+ patients through discussions about the cases. Implementing competency-based, practice-oriented education may not immediately result in structural change, but class discussions can help students generate new ideas and practices to apply in the future. The discussions allowed the students to understand the importance of gender and LGBT+ health care issues in psychiatric clinical education.

Differing from the extant literature, this study revealed the thoughts and feedback of teachers and students on the implementation of the case teaching method and expanded our understanding of the application of case-based teaching. Because of the complexity and diversity of the narrative cases, this study suggests that setting clear, outcome-oriented teaching goals would be a better strategy to produce excellent learning outcomes. With respect to teaching and learning outcomes, inviting LGBT+ to serve as spokespersons in medical education and curricula is highly recommended. 

This study also suggests that educational institutions should implement supportive structures, including the development of a new teaching culture and professional learning, to address the problems faced by teachers in the implementation of case-based teaching. In addition, in terms of research design, if this study could collect students’ opinions and feedback with a simple questionnaire at the end of the course, it might be able to gather more students’ views on case-based teaching, enrich the research data, and increase understanding of the application of case-based teaching.

Overall, the results of this study revealed that narrative cases and case-based learning allowed students to transform their knowledge of gender and their attitudes into concrete actions and LGBT+-friendly medical treatment. This study can provide medical teachers with the means to cultivate students’ LGBT+ competency and incorporate topics related to gender into psychiatric clinical education. The incorporation of case-based teaching into psychiatric clinical education allows medical students to gain a deep understanding of gender in medical and health care and to apply such insights to clinical practice.

## Figures and Tables

**Table 1:** Background information on the participants.

Participants	Gender		Years of Study		Age Range	In Total
Students	Male	Female	Fifth year	Sixth year	23–30	19
	11	8	11	8	19	
Teachers	2	0				2
